# The Role of Foley Catheter Coated with Gold, Silver, and Palladium in Decreasing Urinary Tract Infections in the Intensive Care Unit; a Letter to Editor

**Published:** 2019-07-27

**Authors:** Seyed Hossein Ardehali, Maryam Sedaghatmanesh, Alireza Fatemi

**Affiliations:** 1 Department of Anesthesiology and Critical Care, Shohadaye Tajrish Hospital, Shahid Beheshti University of Medical Sciences, Tehran, Iran.; 2 Internal Medicine Department, Shohadaye Tajrish Hospital, Faculty of Medicine, Shahid Beheshti University of Medical Sciences, Tehran, Iran.; 3 Men's Health and Reproductive Health Research Center, Shahid Beheshti University of Medical Sciences, Tehran, Iran.

**Keywords:** Cross Infection, Infection Control, Intensive Care Units, Sepsis, Urinary Catheters

**Dear editor**

Urinary tract infections (UTIs) are among the most common hospital-acquired infections, which are caused by urinary catheterization in most cases (1, 2). These infections, like other hospital-acquired infections, elongate the duration of hospitalization and can act as a depot for antibiotic-resistant bacteria. For each day that the catheter remains in the urinary tract, the probability of UTI increases by 3% to 7% (3). In 2011, the Centers for Disease Control and Prevention (CDC) reported the rate of catheter-related UTI in the intensive care unit (ICU) as 1.2 to 4.5 cases per 1000 catheters per day and the mortality rate related to these infections has been estimated to be about 15% (4).

Various approaches have been proposed for decreasing these infections. Among these approaches, using aseptic methods, limiting the use of catheters to necessary cases, using disposable tools, and training the personnel can be pointed out. One of the most recent methods suggested in this regard is using catheters coated with anti-septic agents, antibiotics, and agents and metals such as silver, silicon, nitrofurazone (5, 6).

In a study in 24 hospitals, using catheters coated with silicone-nitrofurazone compounds led to a slight decrease in UTI rate with odds ratio of 0.68 (5). In another study in the United Kingdom, the decreasing effect of nitrofurazone-coated catheters on UTIs caused by Foley catheters was confirmed (6). A study on 116 patients in Spain revealed 38% decrease in the rate of UTIs caused by catheters in urinary tract when using catheters coated by gold, silver, and palladium (7).

Fortunately, this kind of catheter has become available to the physicians in Iran. However, since enough experience does not exist regarding their possible harms and benefits in the Iranian population, the researchers of the present study attempted to design a case-control study for evaluating the effects of using urinary catheters coated with gold, silver, and palladium on the rate of infections and mortality due to UTIs in patients hospitalized in the ICU of a teaching hospital in Tehran, Iran.

Inclusion criteria were age over 18 years and having a catheter for 2 to 7 days. Presence of any infection or antibiotic consumption at the time of catheterization or development of an infection not related to the catheter during the study, the patient being pregnant or lactating, and having a history of allergy to the metal compounds of the catheter were among the exclusion criteria. Positive urine culture was defined as presence of at least 100000 bacterial colonies in each milliliter.

Finally, 314 patients were randomly divided into 2 groups of 157 and catheterized with either regular or metal coated catheters. The 2 groups were similar regarding sex distribution (p = 0.518). Mean age of the patients was 59.80 ± 19.85 years in those with metal coated catheters and 61.69 ± 19.57 years in the regular catheter group (p = 0.396). Throughout the study, 26 (16.6%) patients in the coated catheter group developed symptoms related with UTI due to catheter, 18 (11.4%) of which had positive urine cultures (9 cases of Klebsiella pneumoniae, 3 cases of Escherichia coli, 4 cases of Pseudomonas aeruginosa, and 2 cases of Klebsiella oxytoca). All the mentioned microorganisms were resistant to fluoroquinolone family, aminoglycosides, penicillin, third and fourth generation cephalosporins, carbapenems and colistin. 

In the non-coated group, 38 (24.2%) patients developed symptoms related to UTI due to catheterization, 29 (18.5%) of which were reported as positive (17 cases of Klebsiella pneumoniae, 7 cases of Escherichia coli, and 5 cases of Pseudomonas aeruginosa). All the mentioned microorganisms were resistant to fluoroquinolone family, aminoglycosides, penicillin, third and fourth generation cephalosporins, carbapenems and colistin. 

There was no statistically significant difference between the 2 groups regarding developing UTI (p = 0.134). Additionally, no significant difference was found between the coated and non-coated groups regarding infection-related mortality (6 (3.8%) patients in the coated groups vs. 11 (7.0%) in the non-coated group; p > 0.05).

Based on the findings of the present study, although the group using heavy metal coated urinary catheters had a lower number of infections and mortality cases, this difference was not statistically significant. Therefore, we should be cautious regarding suggesting their use considering the price of these catheters and their availability in hospitals. It seems that more accurate and multicenter studies with controlling other probable confounding factors are required for reaching a final decision regarding the use of this tool.
